# An Exploratory Study of Beryllium and UK Soft Touch Regulation: An Enduring Example of Weaknesses of UK Occupational Health and Safety Governance

**DOI:** 10.3390/ijerph191912771

**Published:** 2022-10-06

**Authors:** Andrew Watterson, Matthias Beck

**Affiliations:** 1 Faculty of Health Sciences and Sport, Stirling University, Stirling FK9 4LA, UK; 2 Department of Management & Marketing, University College Cork, National University of Ireland, T12 K8F Cork, Ireland

**Keywords:** UK health, safety regulation, beryllium toxicity, better regulation

## Abstract

Smart regulation, better regulation, responsive regulation, business-friendly regulation and voluntary ‘self-regulation’ have their origins deeply embedded in UK policies in the 20th century. Their aim generally is to reduce workplace regulatory obligations on employers. This can overtly or covertly undermine efforts to improve working conditions. In the UK, the historical control and regulation of beryllium (a toxic metal used in industry) illustrates this problem, and as we illustrate through an exploratory analysis of original archival material and official publications. Soft touch regulation of the metal beryllium was developed within the UK semiconductor industry when tighter controls were proposed in the 1960s and 1970s. Historical industry, government and science responses to health and safety information about beryllium provide important lessons for current debates on occupational health and safety.

## 1. Introduction

Global occupational health history frequently follows ‘macro-patterns’ where tensions are played out between technological innovations, the state of scientific knowledge about hazards and the perceived needs of markets. New diseases caused by work hazards, meanwhile, often remain undiagnosed, unrecorded or unrecognized for long periods before action is taken [1].

The latency period of occupational diseases can be decades. Hence, once a disease is recognized, industrial processes may have moved on or out of a country, and companies may have closed down while affected employees die uncompensated [2]. Where there is evidence of diseases, government compensation schemes, industry lawyers and insurance companies all too often contest scientific findings [3]. Sometimes, regulation comes in after hazardous industries have departed. For example, oil-related carcinogens which caused UK mule spinners’ cancer were outlawed when the process no longer existed in the UK and the retired workers were dying from cancer [4,5].

Historical circumstances can shape occupational health practice and policy in complex and contradictory ways. One example of this is the case of beryllium use and regulation in the UK.

This paper aims to explore archival materials and other secondary sources on beryllium toxicity as a means to understand the formation and application of contemporary approaches to UK occupational health and safety. The objective is to locate, identify and describe some of the factors that contributed to a situation where relatively wide-spread beryllium use paralleled an ability of capital to ‘control’ government, labor, communities, science and policy for both profit and at times for purposes of defense and national security. The study also seeks to identify where exploratory case studies of toxin regulation may sit within regulatory theory, but this is not the primary aim of this paper.

At its core, our exploratory beryllium case study seeks to illustrate the complex interaction of factors which gave rise to under-regulation and the neglect of toxic hazards within a broad thrust of capital exerting considerable control on government while government attempted to exclude labor from information and participation in workplace health and safety matters.

Our paper proceeds as follows: Following this introduction, the next section discusses our archival sources and our methodological approach. We then describe beryllium toxicity, followed by a discussion of how medical views evolved on the risks it poses. Next, we discuss barriers to the control of beryllium hazards in the electronics and other industries, with special focus on the industry–defense–science nexus. This is followed by a description of industrial activity and influence relating to beryllium controls in the UK during the 1950s and 1960s and a discussion of the tension between profit and safety in the UK semiconductor industry. The final historical section discusses the continuing UK threat to workers from beryllium. The penultimate section seeks to estimate harm caused by beryllium-related industries in the UK, while the conclusion draws out key lessons.

## 2. Materials and Methods

The study uses an exploratory mixed methods approach involving historiography to inform an evaluation of UK beryllium regulation and then relates this to a wider assessment of UK occupational health and safety policy and regulation.

Our methodology follows models of exploratory historical archival research in occupational health and safety, with a focus on literature centering in this case on the risks posed by the toxin beryllium to workers and communities. We place special emphasis on documents and sources which hailed from official research organizations and agencies and hence influenced policy, or at least had the potential to do so. Simultaneously, we focus on the role of key decision makers and influencers, who did shape, or could have shaped, the face of regulation in this area. This follows the precedent of research in the area as exemplified by, e.g., Bridget Hutter’s seminal 2001 book, *Regulation and Risk,* which similarly emphasizes the role of key official reports and decision makers, albeit that this study was centered on hazardous work activities rather than a specific toxin.

Specifically, we consulted key texts on occupational health in the UK between the 1930s and 2001 to identify both coverage of and further possible sources of UK and European information about beryllium use and toxicity. Sources that shed light on toxicity are cited and referenced in the paper and details of the search strategy and results are provided in the paper.

As part of our analysis, the National Archives catalogues were searched to identify relevant beryllium files for the United Kingdom Atomic Energy Authority (UKAEA, a variety of research institutions, the Medical Research Council (MRC), the Ministry of Defence (MOD), the Department of Labour and the Ministry of Pensions. These were then examined further for files referring to and providing discussion of toxicity of the metal in the workplace and its regulation. In this context, we focused on correspondence between ministers, scientific civil servants, university researchers, factory inspectors, Health and Safety Executive inspectors and the trade unions.

Searches under the heading of UKAEA produced 478 hits with two large files then being examined in great detail as they included berylliosis claims and referred to the use of beryllium in nuclear research. Files from various other research institutes identified in the study produced 28 hits. MRC searches produced 11 hits and included a mixture of correspondence and published papers. Similar types of material were found in the 5 hits for MOD papers. Searches of the Department of Labour papers based on 3 hits produced material from Her Majesty’s Factory Inspectorate including registers up to 1951 and papers prior to and after the establishment of the HSE with a specific file discussing beryllium in the semi-conductor industry.

The inclusion criterion for our searches was that files mentioned the metal in the context of possible human effects. The exclusion criterion applied to files that focused only on commercial or economic matters. During the searches, it became clear that several but not all the archives included copies of journal articles or parliamentary debates on worker compensation examined elsewhere in the study. Similar types of material were found in the five MOD papers.

The potential limits of our exploratory approach were that key documents may have been missed in the searches or that material was withheld from archives or destroyed. However, the strength of this approach arose from the fact that accessing archives not hitherto searched could provide new material to inform assessments of the OHS policy development in the area. This proved to be the case with beryllium.

We also touch on, but do not develop in any great detail, where our exploratory case study sits in the context of regulatory theory, and this is a limitation.

## 3. Analysis

### 3.1. The Properties of Beryllium and Its Risk Assessment

Beryllium is a hard, strong, light metal with a high melting point and properties of value to the engineering, health, nuclear and defense industries. Looking at risks of this kind in general, Fox describes several ways according to which risks can be perceived: “the first perspective (materialist or realist), maps risk directly onto underlying real hazards. In the second (constructionist or culturalist), views hazards as natural, while risks are social constructions. In the final (postmodern) position, both risks and hazards are seen as constructions [6]. For workplace hazards, neither hazards nor risks are usually fully recognized when manufacturing begins. When an initial recognition of beryllium risks emerged, it was based on a growing ‘natural’ understanding of what the acute human toxicity of beryllium was and not a post-modern view of beryllium. A Bhaskarian critical realist perspective therefore might apply to beryllium [7], in the sense that science gradually demonstrated a variety of hazards posed by beryllium whilst the risk assessment and risk management of the metal remained socially constructed often to benefit capital and the state, but not necessarily in a formulaic way.

UK Beryllium regulation reflected largely what the environmental economist Costanza has described as the workings of ’technological optimists’ as compared to ‘prudent pessimists’’ [8]. Technological optimists displayed a ‘white coat syndrome’—believing to give ‘us…the resources that will solve any problems we have.’ In the electronics, armaments and medicine sectors, the ‘optimistic’ argument was—‘Provide us with a super conductor metal like beryllium and we will be able to use it safely and effectively.’ The parallel assumption was that capital and science should adopt an unfettered pioneering approach to potential and proven hazards in the belief that this would produce the best possible outcome for production and profits. Prudent pessimists, meanwhile, argued, for much greater caution when introducing new materials, for far stronger engineering controls to reduce or remove user exposure and much greater health surveillance and epidemiological surveillance of the exposed workforce [9].

### 3.2. Beryllium Hazards and Risks: The Emergence of Conflicted Medical Views

Beryllium was first discussed in 1799 by French chemist LN Vauquelin. By 1886, Siem had explored beryllium toxicity in animals [10]. In the 1900s, initially inaccurate reports of acute adverse health effects among those mining beryllium appeared. In the 1930s, reports on adverse health effects affecting those manufacturing or processing beryllium products appeared in Germany [11], then Italy [12], and Russia followed [13]. Contact dermatitis was reported in workers exposed to dust and fumes in 1938. In the same year, the ILO Encyclopedia reported Russian clinical beryllium fluoride poisoning cases from 1936 which led to skin and pulmonary problems [14]. In 1940, Berkovitz and Israel described Russian workers poisoned by beryllium and, in 1948, the first UK beryllium fatality was reported, involving a physicist who worked on fluorescent lamps [15]. In the 1940s, laboratory evidence emerged on beryllium carcinogenicity. A US occupational physician noted: “Although the adverse responses to beryllium compounds had been recognized in Germany and the Soviet Union in the 1930s and early 1940s, the relevant reports in the medical literature of these countries were not widely distributed. Beryllium disease, or more accurately beryllium diseases, were recognized in the USA in the early 1940s… [16]”. The knock-on effects of this were significant as the UK relied on US assessments and sources [17].

Some UK and US occupational physicians acknowledged that new materials used in industrial development could became indispensable ‘but their properties may remain for a long time insufficiently understood [18,19,20]. These writers adopted a prudent approach to the metal based on their clinical work. In 1942, Hunter recognized problems associated with beryllium in relation to copper–beryllium alloys in aircraft construction and noted that the manufacture of beryllium steel produced beryllium oxide and beryllium fluoride dust linked to molten salt electrolytic baths. He also noted that ‘the dusts of all these compounds are known to be toxic to animals’ [21]. As Head of the MRC occupational medicine unit at London Hospital, Hunter was one of the first UK physicians to identify adverse health effects of beryllium. Nonetheless, he supported industry in contesting beryllium poisoning cases as a medical consultant for Johnson Matthey, beryllium producers in the 1960s [22]. Researchers working with Hunter, such as John Agate, expressed concerns following the diagnosis of beryllium poisoning cases in 1948: “Where possible, substitution of other compounds in place of those of beryllium is clearly the best preventive measure. In processes where this element must still be used, vigorous suppression of all dust and fumes, and regular medical supervision of workers are necessary” [23]. Agate visited the USA in the 1940s to discuss the occupational hazards of beryllium and attended a conference there on the metal.

However, a 1951 commentary in the Lancet adopted an extreme technological optimist perspective, stating that: “Beryllium seems to be the Admiral Crichton of metals. It is nearly as light as magnesium and is more elastic than steel. It is strong and hard and it resists heat and corrosion. Under nuclear bombardment it is a most efficient source of neutrons [24] “… to charge such an admirable metal with having poisonous properties is about as distasteful as accusing a trusted butler of stealing the family plate. The story of beryllium is ‘… fascinating and contradictory. Few people are completely sure that beryllium is the sole cause of illnesses ascribed to it.”

This reflected less science and more faith than perhaps was acceptable in a medical journal even in the 1950s. By 1953, leading UK public health physicians like Leff, were aware of the risk of beryllium. Getting an ‘industrial’ disease prescribed required much data and medical consensus yet beryllium was put on the industrial disease schedule Leff noted: “As we obtain more extensive knowledge of the different hazards of work: for instance, poisoning by beryllium and its compounds, [it was] added in 1949” [25]. What is significant here is that the reporting of some, but not all beryllium-related diseases, progressed over time.

In many respects, however, beryllium disease identification and prevention were slow. This related to a combination of factors. From the 1930s to 1960s, the UK Factories Acts, with duties to prevent exposure to certain dusts and fumes, were viewed as adequate for controlling such hazards. However, these provisions were never widely used by HMFIs (Her Majesty’s Factory Inspectorates). In parallel, there was little published evidence that beryllium poisoned many UK workers and hence controls and working conditions could be seen as adequate [26,27,28].

Papers from studies commissioned by HSE (Health and Safety Executive) and involving staff from HSE in the 1970s and 1980s and other sources, however, disproved this view [29]. Beryllium exposures, below the established threshold limits, were found by this research to present serious disease risks [30]. Beryllium poisoning cases occurred in the ceramic electrics industry but had often not been properly diagnosed, reported or recorded. This may have been due to a lack of knowledge, lack of enforcement staff and also a reluctance on the part of senior HSE staff to seek out occupational disease problems because it would necessitate intervention in the important defense, medical and high tech industries. Alice Hamilton, a leading independent occupational physician in the US, considered the identification of beryllium toxicity in 1948 to be a success for toxicology and occupational medicine: “To anyone, who like me, can look back to the early days of industrial medicine, this story of beryllium poisoning is an amazing thing. Here in the space of some six or eight years, a form of industrial poisoning that was highly dubious, involving only a small number of cases, not included under compensation laws except in full-coverage states, observed by a small number of physicians, was promptly made the subject of thorough study will full publicity … When I look back on the days when we were urged not to mention such a thing as TNT poisoning lest we drive all workers out of the plants, I can hardly believe it is the same country” [31].

In some respects, the US story was a success, certainly compared to the control of many other toxins discussed by Hamilton. However, beryllium presented a range of occupational disease challenges, including potentially fatal acute threats and more serious chronic effects from very low-level exposures. Not all were dealt with as effectively as Hamilton thought. The acute disease threat from a high-level short-term exposure to beryllium, so-called ‘berylliosis’, was rapidly fatal. The chronic disease, now referred to as chronic beryllium disease (CBD) and described in the 1950s, was typically not recognized or frequently misdiagnosed. Craig Zwerdling provided an account of such misdiagnosis in the ‘Salem Sarcoid’ cases [32]. Beryllium disease cases were at times described and diagnosed as TB cases. The relationship between the company using beryllium and some of the researchers investigating the cases was flawed and worked against the workforce. The carcinogenicity of beryllium took a long time to be accepted despite lab tests that indicated such effects in the 1940s [33,34,35].

### 3.3. Barriers to the Control of Beryllium Hazards in the Electronics and Other Industries: The Industry–Defense–Science Nexus

Several factors influenced the decision to use beryllium in commercial, medical and defense products. One key factor appears to have been benefits brought to product quality and hence sales and profits. Decisions were aided by a lack of independent research, and a failure to act on, or disseminate, research that had been done, as US commentators as Egilman and others have flagged up [36,37,38,39]. In the UK, it related predominantly to market and national political interests—defense, arms sales, shareholder interests, plus industry framing, conducting, controlling, reporting and validating research.

**Figure 1:**
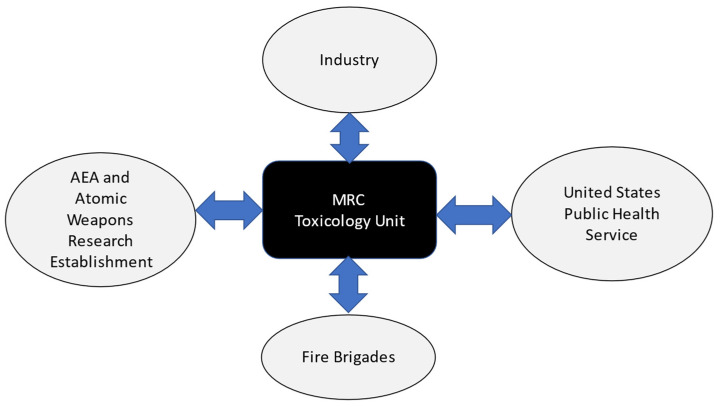
MRC toxicology unit links with UK and US agencies and industry. UK medical research council toxicology unit links with UK and US agencies.

**Figure 2:**
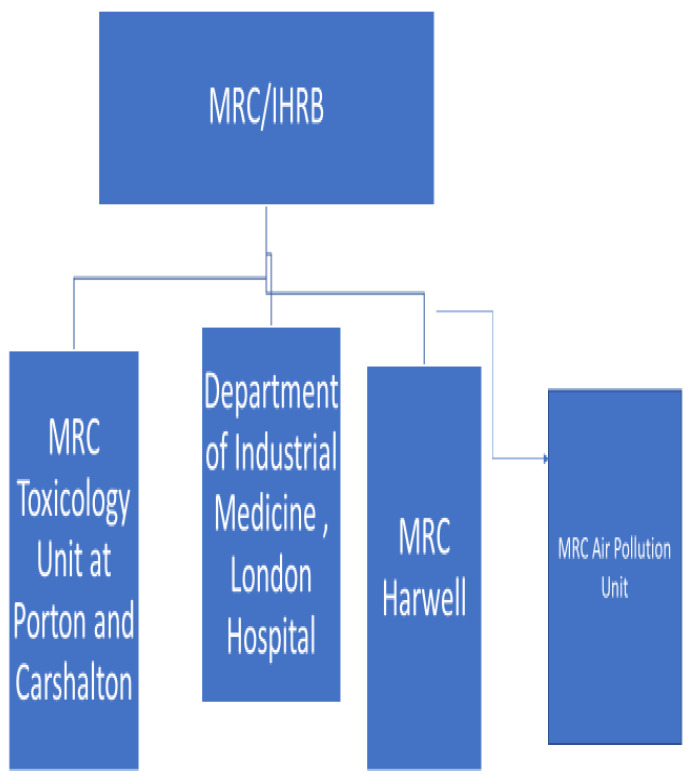
MRC links with atomic weapons and biological warfare bodies. UK medical research council/industrial health research board links with atomic weapons and biological warfare bodies.

Some of those making beryllium in the early days had ironically displayed greater prudence than those researching its toxicity in later years. Some beryllium companies were all too aware of the acute and chronic human health risks from all forms of the metal, having witnessed the illnesses and being exposed to the risks as managers and owners when in their own factories [40,41]. Leading toxicologists in UK research institutes seemed to have trusted US reports on the safety of beryllium uncritically. These reports neither reflected European research from the 1930s and 1940s nor the mortality reports from the USA and UK from the. Some government scientists expressed concerns about beryllium carcinogenicity, but no action followed [41]. This seems to have at times related to ‘scientists’’ club’ mentality where results of other scientists were rarely questioned as well as, during the immediate post war period, the close relationships between government toxicologists, the atomic weapons industry, US agencies and even some leading occupational physicians. We illustrate this schematically in Figure 1 and Figure 2 below [41].

Barnes at the MRC Toxicology Unit based in Carshalton and Porton—the germ warfare research center of the UK—acknowledged that he lacked experience of industrial toxicology. He accepted the USPHS (US Public Health Service) line that beryllium itself was not toxic and told the UK industry so, explaining some illnesses on the basis of exposure to other substances [41].

### 3.4. Industrial Activity and Influence Relating to Beryllium Controls: 1950s and 1960s

In the 1950s, the UK Atomic Energy Authority needed beryllium. The UK company ICI and others in the UK produced that beryllium. ICI did tests on background beryllium levels near a planned plant and found that beryllium levels were ten times higher than US permitted levels (0.01 µg/m^3^). The MRC Radiobiology unit’s view was that US standards had been produced ‘perhaps in panic’ [22]. Differences in views reflected differences in the method of obtaining scientific results, their interpretation or in terms wider risk assessments. The role of the UK Ministry of Defense is not fully clear, but we know about cooperation between the MOD and the Atomic Weapons Research Establishment. There were also close links between the MRC Harwell units and the MRC units at Porton and the London Hospital with Hunter appearing in court as defense witnesses for the beryllium industry. The extent to which such links might have informed, influenced or skewed risk assessments and health and safety regulation is not fully known, but there are indications that the risks from materials such as beryllium were systematically played down by all three MRC units [40].

Among those who disagreed, one of Her Majesty’s Factory Inspectors (HMFIs), Brian Harvey, noted that: “Substitution is to be preferred to other methods where the process in question is highly dangerous or materials are particularly toxic. In these cases, it offers the only commercially practicable solution to the problems. Even where expensive research is necessary, it may still be a cheaper method in the long run” [42]. This view differed from the ILO Encyclopedia on Occupational Safety and Health for 1961, which advocated a less rigorous exposure approach [10]. In 1958, Harvey stated in relation to the fluorescent lighting industry, that: “beryllium is so highly toxic that there is reason to suppose the precautions necessary to make safe its use might have been financially crippling” [42].

The British Chemist DA Everest, a true technological optimist, stated in 1964 that: ‘the safety rules are carefully followed the risk [from beryllium] is infinitesimal’; ‘usually laboratories handling beryllium have been designed under expert guidance and the personnel are subject to regular medical examination’ [43]. Compared to laboratory contexts, the possibility of applying such controls to foundry or engineering works were more limited. Everest was familiar with the scientific literature and knew that, in 1936, Gelman in Russia recommended beryllium be machined only in well ventilated rooms with gloves and other protective clothing provided and washing accommodation available.

Everest acknowledged only two UK beryllium fatalities which had occurred before 1949, but he was aware that in the neighborhood of 47 were ‘well established’ in the US [43]. Everest promulgated the US beryllium worker selection criteria that Tepper and others had produced: namely, that personnel should be selected on the basis of ‘medical history, a physical examination and a chest X-ray. These examinations should reject those with a greater than average chance of developing beryllium disease on account of suffering from chronic respiratory diseases or from skin diseases which might make them susceptible to beryllium dermatitis”. Those with asthma, heart disease, TB and abnormal chest X-rays were also rejected [43]. Existing CBD cases were not necessarily being picked up, and Everest appears to have assumed that good lab practice would protect workers from exposures. They did not. Nor could the selection criteria protect workers from chronic diseases and cancer as some of the literature and research conducted at the MRC by Barnes [34] was already revealing.

The metal became common in British industry between 1940 and 1960 [44]. In the early 1960s, beryllium was used as alloy of copper, nickel and aluminum; as deoxidizer in steel making where dust was given off from electrolytic baths; as rods to work graphite piles in the atomic energy industry; as transparent foil in industrial X-ray tube windows; as powdered phosphors in fluorescent strip and electrical sign lamps and tubes; and as crystals in radios and as electrical porcelain [4].

A frequent paradox applied to beryllium processing was the assumption that, if a substance is safe, it should be safe for any worker. This was not what was being suggested, however, because reference was made to average chances of contracting a disease as well as to medical screening. The evidence base for making such fine calculations was non-existent at the time, and the approach could not ensure worker safety. Everest stated that the existing UK beryllium standard had ‘no sound scientific basis and is probably highly conservative’ [43]. This was crude technological optimism with no signs of the precautionary or prudent principle in sight.

### 3.5. The Development of the Semiconductor Industry

Great Britain was a contributor to the growth of semiconductor and microelectronics technologies in the 1930s. After 1945, the UK “depended for the most part on American semiconductor developments and assumed the role of importer rather than exporter of ideas” [45]. However, companies like Mallards were only a few years behind the USA in the 1950s. Practice evolved from the use of materials and production methods developed in other industries, especially in the defense industry and in medical research. In the 1940s and 1950s, electronics were considered a cutting-edge industry. Some commentators argued that, as such, the industry’s capacity to identify, remove or reduce exposures of employees to hazardous substances was enormous [46]. Meanwhile, some of the hazards of the electronics industry were well recognized and involved what were described as ‘cutting edge’ materials or processes. Solvents were widely used and so was radiation, glycol ethers and a variety of chemicals based on metals such as arsenic and beryllium: which were known carcinogens [47,48,49].

Nonetheless, a mythology was created that the electronics industry in general and the semiconductor industry and chip manufacture in particular were ‘clean’ and safe industries [50,51,52,53]. This related to innovative manufacturing technologies and engineering techniques and the rate of change in the industry [54]. This position was re-enforced by confusion about chip production. Chip production requires a ‘clean’ environment to ensure the quality of the product. This does not mean that such an electronics environment is a clean and healthy one for employees [55,56,57]: indeed, the system of ventilation employed may make the working environment potentially more hazardous to workers [58].

The ‘high tech’ Image of the industry made it difficult to question the specialist expertise of the industry. This included its assumed capacity to protect workers from short- and long-term hazards as well as its ability to provide auditable evidence on the state of occupational health and safety in plants. Additional complications arose from the fact that major transnational companies often employed more resources in relation to occupational medicine and hygiene matters than national governmental regulatory and inspection agencies [59].

The export of outdated technologies and materials in industries such as electronics to less well-regulated parts of the globe caused further problems [60]. For the electronics industry, these areas often offered investment grants, promises of light touch environmental and health and safety controls, and a pliant but well-trained workforce [61]. Accordingly, the semiconductor industry moved out of North America and Western Europe to Asia and Eastern Europe [49] and also more peripheral areas of the UK, such as Scotland and Wales. IBM had come to Scotland after 1945 and made punched card sorters followed by typewriters at Greenock in the 1970s and then computers in the 1980s. National Semiconductors, an American company, came to Greenock in Scotland in the 1970s (NSC web page), and Motorola also appeared in Scotland in the 1970s and 1980s. In the 1950s, the US firms Texas Instruments, SGS Fairchild and Hughes Semiconductors opened facilities in the UK. Philips entered into a research development agreement with Philco (the Philadelphia Battery Company) in 1957.

Linked to the development of transistors, the Electronic Valve and Semiconductor Manufacturers’ Association (VASCA), was set up in the UK in 1959. VASCA played a leading part in ensuring the soft regulation of beryllium in the industry.

### 3.6. Profit and Health and Safety Policy in UK Semiconductors

The attitude to beryllium in the UK was colored by specific ideological, cultural, political and economic influences. The UK approach to beryllium toxicity and diseases during the 1940s to 1960 matches a critical realist interpretation—hazards are sometimes recognized but risk assessment and risk management are skewed in favor of the perceived needs of powerful interests.

The semiconductor industry expressed concern about HMFI enforcement variations on beryllium controls in the late 1960s. VASCA argued for a code of practice, while seeking to avoid prescriptive regulations. All valve manufacturers were members of VASCA, but there were several major employers and a further 60 smaller firms manufacturing semi-conductors who were not members of VASCA. A working group involving VASCA and the Ministry of Labour (including HMFIs and chaired by Harvey) was set up to produce a code of practice. Industry did not want workers or their unions involved as the code would be ‘entirely technical’, and they pressed for less rigorous wording: especially lobbying for ‘should’ to replace ‘shall’ [62]. HMFI accepted the exclusion of trade unions from the discussions.

In 1972, these deliberations resulted in the Department of Employment HM Factory Inspectorate Code of Practice for the use of beryllia ceramics and a memorandum of guidance to medical officers. The Code followed an approach to the Department by the Electronic Valve and Semi-Conductor Manufacturers’ Association (VASCA) for advice on precautions connected with the handling of articles containing beryllia. Accordingly, the code was: “Advisory only and has no legal sanction. It is intended to apply to the use of beryllia in factories engaged in the manufacture of valve and semi-conductor components but there is nothing in the code which could not be applied in other industries handling beryllia”.

The major impact of the industry in replacing ‘shall’ with ‘should’ in the code is shown in bold in Box 1 below.

Box 1 **HMF1 Code of Practice for beryllia ceramics 1972 caption.**

’Material or components received **should** have a surface dust level not exceeding 0.01 micrograms per square centimeter. The exposure area **should** be segregated as far as practicable so as to limit the number of persons exposed. As far as practicable only designated personnel **should** enter a beryllium exposure area. Exhaust ventilation equipment or other equally effective equipment shall be provided where necessary to control the beryllia or beryllium compounds in the air of any workplace to below the levels set out … The exhaust ventilation equipment or alternative equipment if used **should** be examined and tested at frequent intervals by a competent person and a record of such examination and testing **should** be kept and certified by a responsible person. Cleaning equipment used for removing beryllia contamination **should** be such that hazardous amounts of beryllia dust above the limits did not escape. Safe procedures **should** be devised for removing beryllia which has inadvertently escaped during processing. A record of any such occurrence **should** be kept together with details in accordance with Part II, para 16. A competent person **should** be informed if any beryllia component is lost or damaged. Persons leaving the industry **should** be given a card recording that there has been occupational exposure to beryllium, to give to the family doctor.’

Thus, powerful industry intervention weakened a code of practice which in itself was already a weak control or enforcement tool. Despite the long history of ill-health associated with beryllium exposure, despite early industry worries about the toxicity of beryllium dusts based on their practical knowledge, despite sound principles of engineering action and product substitution identifying means to remove employee exposure to beryllium dust and fumes, and despite the early advocacy of a precautionary policy by public health professionals to beryllium—warnings were watered down or disregarded. This followed a report from 1964, when medical researchers in Wales had identified two industrial ceramics workers out of a workforce of 130 with CBD with a third case in a smelter and followed them for 10 years [63].

### 3.7. The Continuing UK Threat from Beryllium

The use of beryllium in UK industry increased, and medical researchers recognized that UK occupations at risk included not only metal workers and ceramic manufactures but also those in the electronics industry—TV manufacture, transistors and heat sinks—and those working in scrap metal disposal. Women, especially pregnant women, were categorized as at special risk. The fatality rate for all workers with Chronic Beryllium Disease (CBD) was around 35%, so beryllium poisoning was clearly a major threat [64]. By 1973, the pathologist Jones Williams and colleagues had found, through in vitro testing, that 7 of 50 healthy beryllium workers were sensitized to beryllium [64]. Two apparently acute cases of beryllium disease were reported in the UK. The diagnosis was prefaced by a qualifying remark that each worker could already have had CBD as each had worked for several years with the metal. Each case followed a high exposure—one of a worker smelting beryllium in a factory where a batch had apparently been wrongly labelled: the other in a factory where there had been a breakdown in a furnace extraction process [65]. Beryllium usage in the electronics industry continued into the 1980s as a metal dopant in wafer fabrication and plating assembly and into the 1990s in ceramic packages where the metal is used as an alloying agent.

## 4. Discussion

Estimates of CBD (Chronic Beryllium disease) in work in the 1990s–2001 ran at 2–15% at 2 µg/m^3^ level, depending on jobs being done [48,66]. In the UK, 250 workers were thought to be continuously exposed; 1000 workers were occasionally exposed to ‘very low concentrations of beryllium or beryllium oxide’ (HSE statement 2003 although again the detailed evidence base for these estimates of exposed employees is unavailable). Using the estimate of 2% of exposed beryllium workers getting CBD, in the UK, at least 4.5 cases would have been expected. Between 1994 and 2002, HSE recorded one case indicating 3.5 more cases probably occurred in the 250 workers and an unknown number in the 1000 workers. This excludes workers exposed to several alloys and other sources of beryllium. Between 1945 and 1985, the UK beryllium register recorded 49 cases of CBD and 21 of these had died from respiratory failure [67,68]. Between 1985 and 1988, nine new CBD cases were reported [67,69]. However, many cases of beryllium-related disease in a Welsh electrical ceramics factory occurred but appear never to have been properly recorded [48]. Information supplied to UK semiconductor workers on beryllium carcinogenicity remained poor into the 2000s [70]. Accurate cancer estimates from occupational and environmental beryllium exposure are lacking, reflecting similar problems encountered for example in assessing worker exposure to asbestos [71].

By 1985, both the USA and UK had similar and weaker policies on beryllium, as a probable human carcinogen, than either West Germany or France. The latter countries placed restrictions on the metal and insisted on monitoring [72,73]. This fits with a general failure to regulate occupational health and safety effectively within the UK [74] and specifically within the UK semiconductor industry [49,57]. Into the 21st century, many beryllium hazards such as carcinogenicity and low level chronic hazards remained neglected or undocumented [57]. Today, the global beryllium market continues to grow with the US and Europe being the largest consumers of the metal [75].

## 5. Conclusions

The study focused on how toxicological findings and gaps in relation to beryllium were used to inform and sometimes also to bend UK regulatory within a context that had political and ideological roots [76]. How such political decisions could be located within the broader field of regulatory theory is not explored in detail in this exploratory paper. Ayres and Braithwaite theorized that those responsible for governance would be responsive to the regulatory environment and to the conduct of the regulated in deciding whether a more or less interventionist response is needed. Thus, if technology was likely to resolve an issue soon, employers were responsible or employees protected themselves through cautious working practices, and no regulation should be introduced, otherwise regulatory intervention is called for [77]. In the case of beryllium, technology did not resolve major risks, yet effective regulatory intervention did not emerge either.

The problematic politics of occupational health and safety in the UK have been carefully reviewed by a number of authors, including most recently by James and Walters [78]. These authors explore how, starting with the 1972 report of the Committee of Inquiry into Safety and Health at work chaired by Lord Alfred Robens, UK governments sought to: ’shift governance and regulation of work health and safety away from prescription and towards a more principle and process-based approach’. This resulted in the creation of ‘a number of industry and subject-based Joint Advisory Committees’ where ‘the people who created risks and those who worked with them would be involved in decision-making’. Much of the tripartite approach depended on strong worker participation and the approach was vulnerable where, as in case of Beryllium, government and employers prioritized their seemingly common interests, over issues of worker safety and occupational health. Problems with this ‘pseudo-tripartite’ approach became more pronounced in the 1990s when union membership declined and a number of reforms sought to reduce ‘regulatory burdens’ faced by employer [79]. Among other issues, these reforms intended to move ACoPs (Approved Codes of Practice) increasingly in the direction of guidance, with only limited obligations on employers. Woolfson and Beck document how the 2007 election of the New Labour Government in 2007 did little to reverse these trajectories [80]. Instead, New Labour supported a new rhetoric around the notion of ‘better regulation’, which sought to frame the need to protect workers from health risks in a weak and questionable consensualist framework.

Our analysis in this sense is consistent with recent critical analyses of the UK regulatory approach [78]. We question the myth of an identity of interest between employer, regulator and employee on beryllium occupational health and safety controls. We also identify weaknesses in early UK regulatory standards and discuss how those flaws might have rendered regulatory standards especially vulnerable to manipulation by a succession of governments espousing neo-liberal political ideologies.

For beryllium, the pairing of state and employer interest around what was perceived as the frontier industry of electronics appears to have undermined regulatory stringency as did a more recent phase, where health and safety regulation was often perceived as a ‘burden’. Added to this was an overly optimistic attitude of scientists who often prioritized the development of new technologies and over-estimated the applicability of technical protective measures. Combined with an industry which frequently saw more appropriate and cautious health and safety measures around the use of the metal as a potential danger to its profitability, this allowed for what one could describe as wide-spread trivialization of the dangers of beryllium use. One of the most worrying aspects of our analysis is the possibility that there are other, largely undetected technical or chemical hazards, which might have been under-researched and under-detected: recent research even revealed dangerous levels of beryllium in concrete [81]. In the UK, post-Brexit, ineffective regulation and possibly weak self-regulation of such hazards in the workplace may become even more extensive [82].

## Data Availability

The UK National Archives at Kew, Richmond, TW9 4DU hold the UK Government and agency files that were open to the public on beryllium and cited in this paper. Public access to some papers may still be restricted. Hunter MRC and other papers and publications on beryllium can also be accessed at the Wellcome Collection, 183 Euston Rd, London NW1 2BE.
